# Supplementary material for: Effect of Combined Antiretroviral Therapy on the Levels of Selected Parameters Reflecting Metabolic and Inflammatory Disturbances in HIV-Infected Patients
Source: J Clin Med. 2022 Mar 19;11(6):1713. doi: 10.3390/jcm11061713 (PMC8954290; doi:10.3390/jcm11061713)
Supplement: Supplementary file 1 [file jcm-11-01713-s001.zip › jcm-1616885-supplementary.pdf]

A

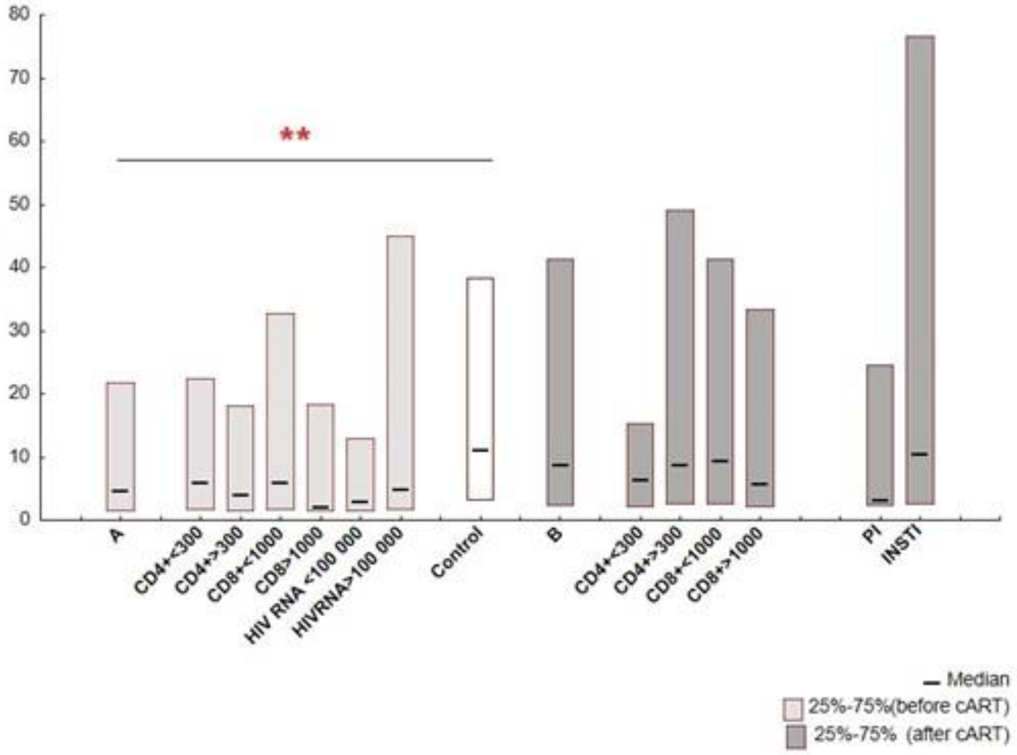

80
70
60
50
40
30
20
10
0
ng/mL
**
A
CD4+<300
CD4+>300
CD8+<1000
CD8>1000
HIV RNA <100 000
HIVRNA>100 000
Control
B
CD4+<300
CD4+>300
CD8+<1000
CD8+>1000
PI
INSTI
Median
25%-75%(before cART)
25%-75% (after cART)

B

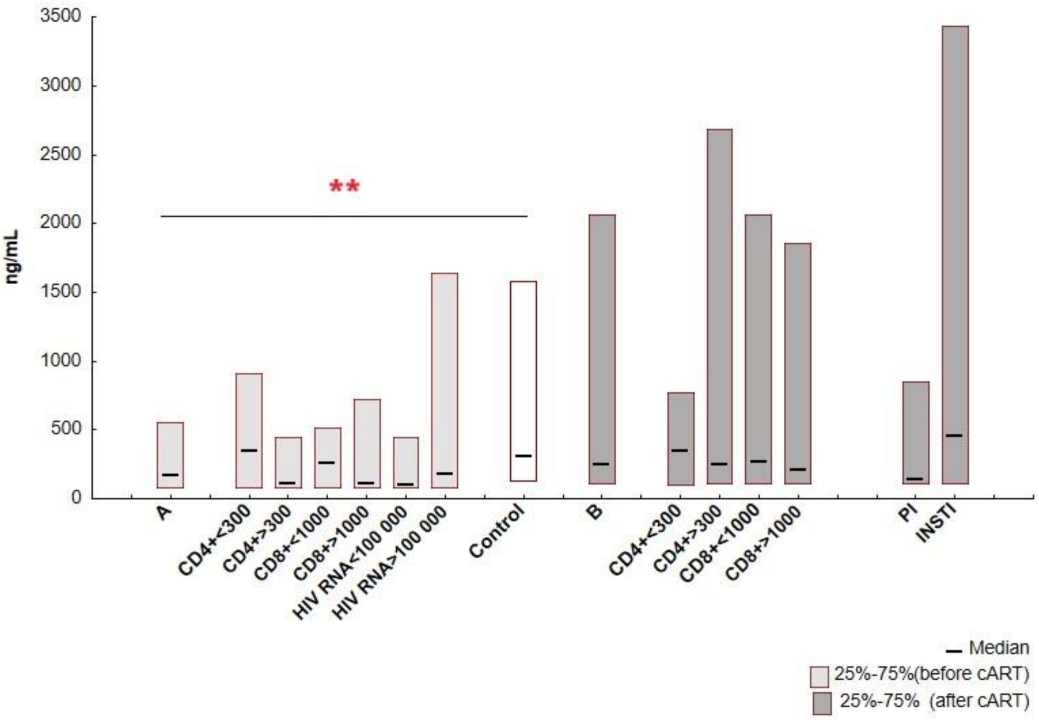

3500
3000
2500
2000
1500
1000
500
0
ng/mL
**
A
CD4+<300
CD4+>300
CD8+<1000
CD8+>1000
HIV RNA<100 000
HIV RNA>100 000
Control
B
CD4+<300
CD4+>300
CD8+<1000
CD8+>1000
PI
INSTI
Median
25%-75%(before cART)
25%-75% (after cART)

C

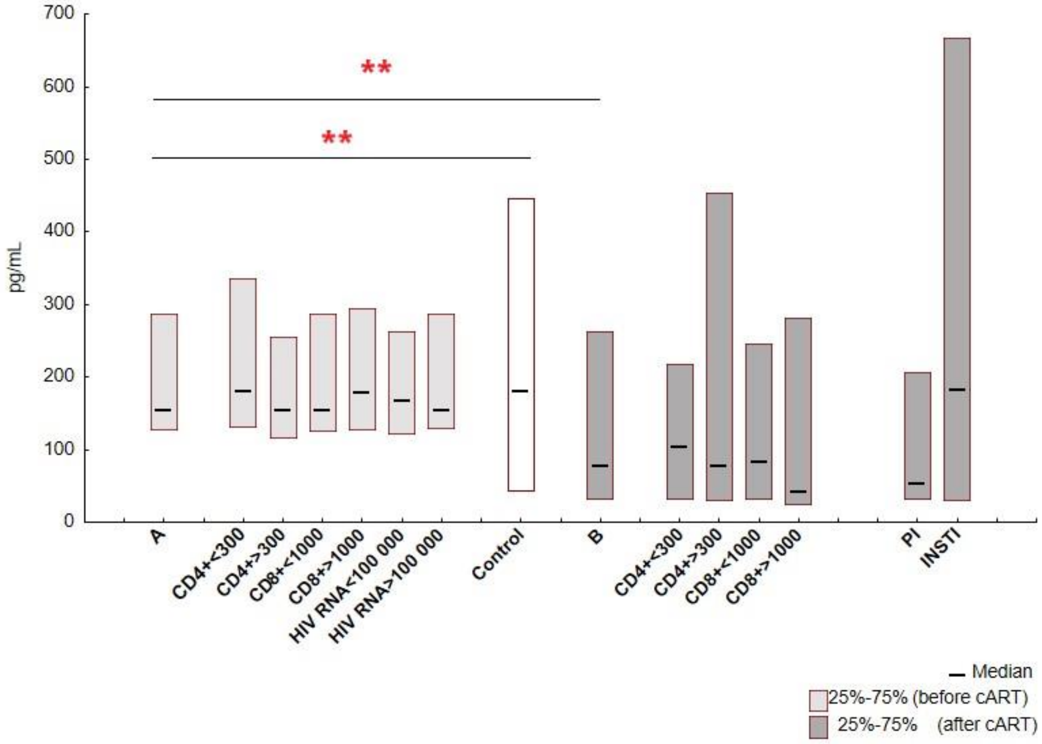

700
600
500
400
300
200
100
0
pg/mL
**
**
A
CD4+<300
CD4+>300
CD8+<1000
CD8+>1000
HIV RNA<100 000
HIV RNA>100 000
Control
B
CD4+<300
CD4+>300
CD8+<1000
CD8+>1000
PI
INSTI
Median
25%-75% (before cART)
25%-75% (after cART)

D

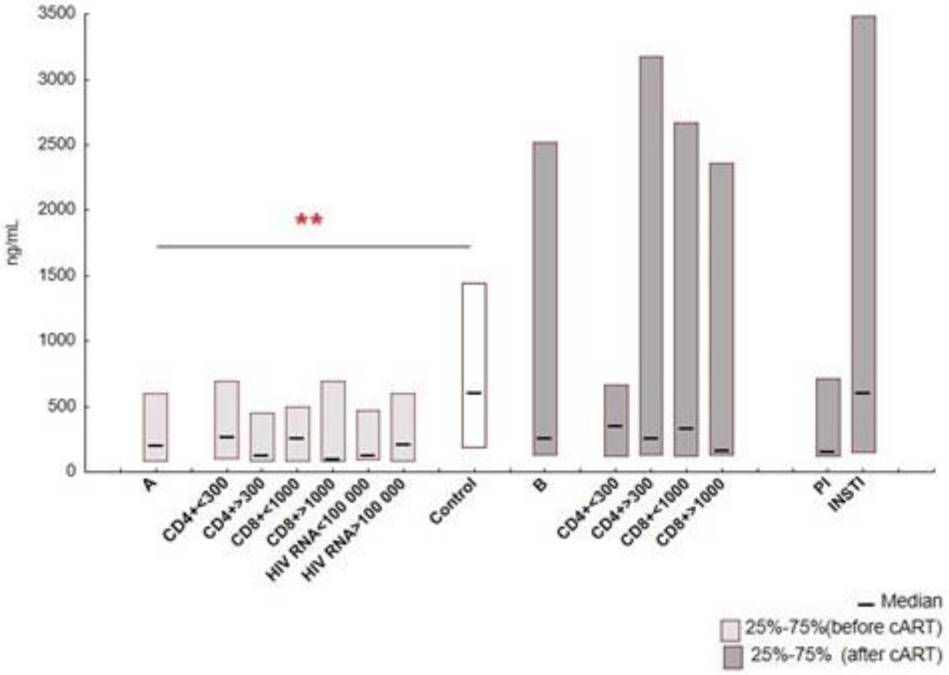

3500
3000
2500
2000
1500
1000
500
0
ng/mL
**
A
CD4+<300
CD4+>300
CD8+<1000
CD8+>1000
HIV RNA<100 000
HIV RNA>100 000
Control
B
CD4+<300
CD4+>300
CD8+<1000
CD8+>1000
PI
INSTI
Median
25%-75%(before cART)
25%-75% (after cART)

E

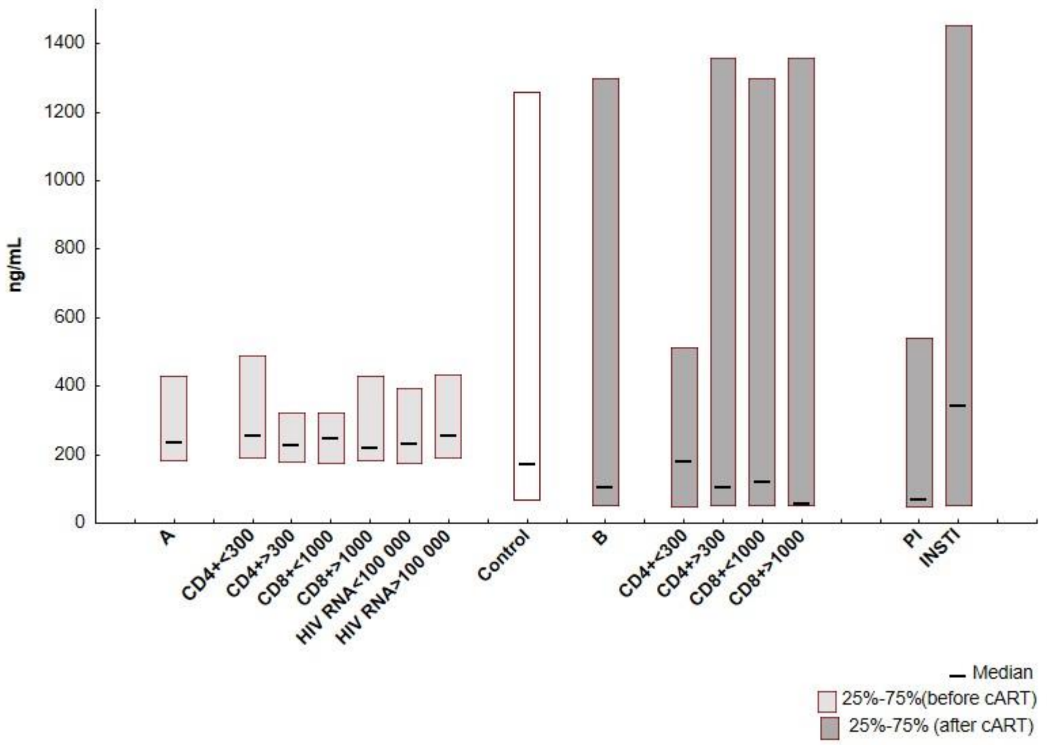
ng/mL
1400
1200
1000
800
600
400
200
0
A
CD4+<300
CD4+>300
CD8+<1000
CD8+>1000
HIV RNA<100 000
HIV RNA>100 000
Control
B
CD4+<300
CD4+>300
CD8+<1000
CD8+>1000
PI
INSTI
Median
25%-75%(before cART)
25%-75% (after cART)

F

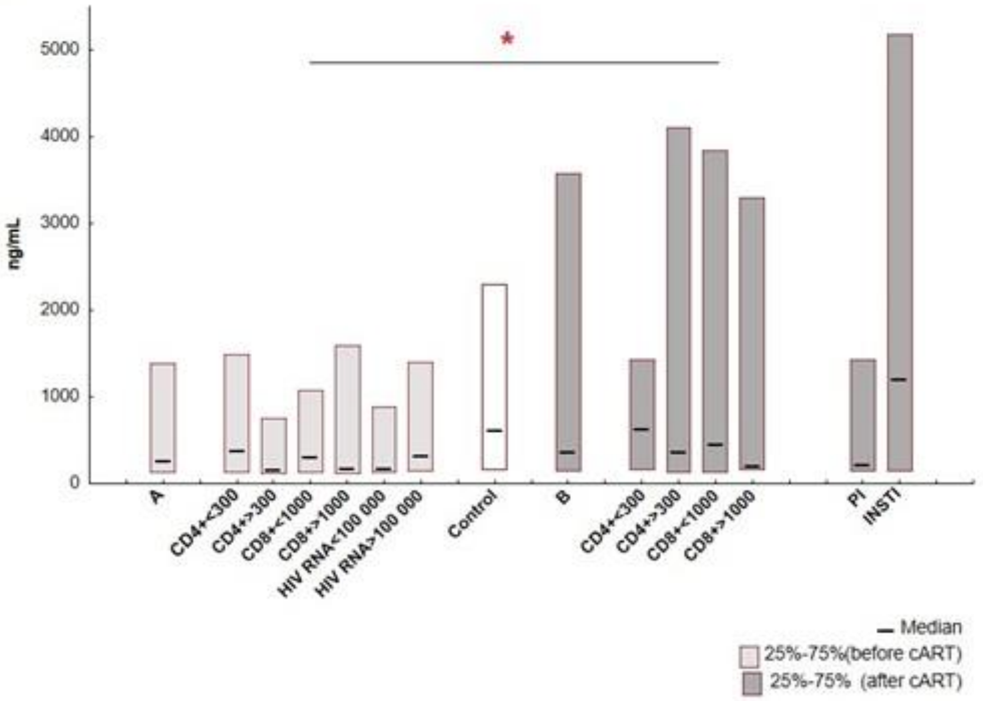
*
ng/mL
5000
4000
3000
2000
1000
0
A
CD4+<300
CD4+>300
CD8+<1000
CD8+>1000
HIV RNA<100 000
HIV RNA>100 000
Control
B
CD4+<300
CD4+>300
CD8+<1000
CD8+>1000
PI
INSTI
Median
25%-75%(before cART)
25%-75% (after cART)

G

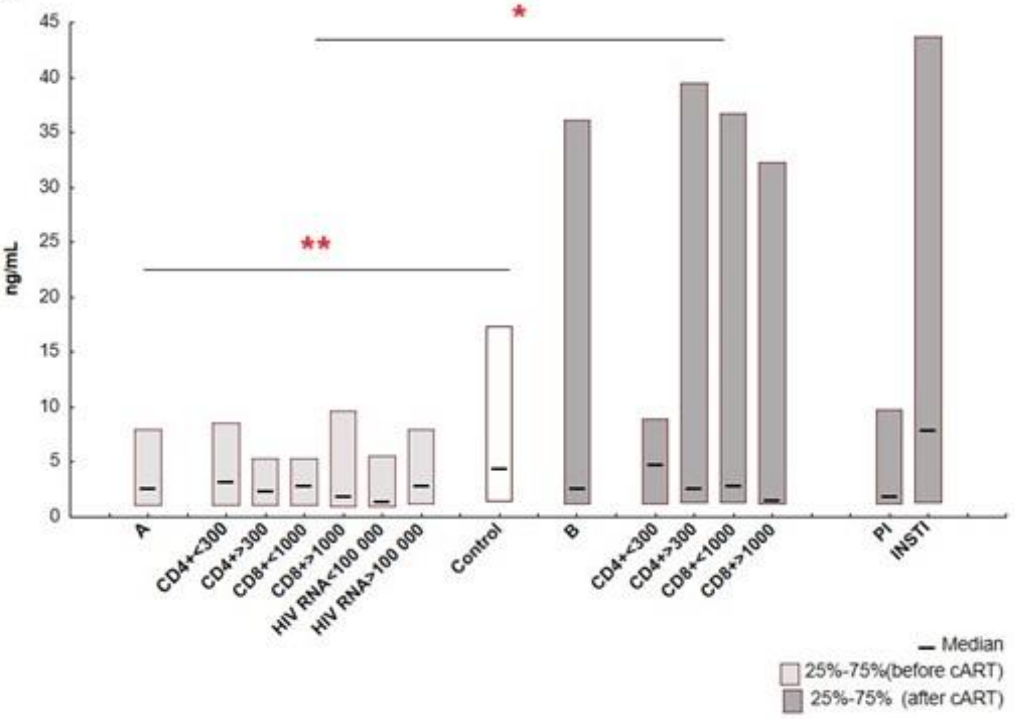
45
40
35
30
25
20
15
10
5
0
ng/mL
**
*
A
CD4+<300
CD4+>300
CD8+<1000
CD8+>1000
HIV RNA<100 000
HIV RNA>100 000
Control
B
CD4+<300
CD4+>300
CD8+<1000
CD8+>1000
PI
INSTI
Median
25%-75%(before cART)
25%-75% (after cART)

H

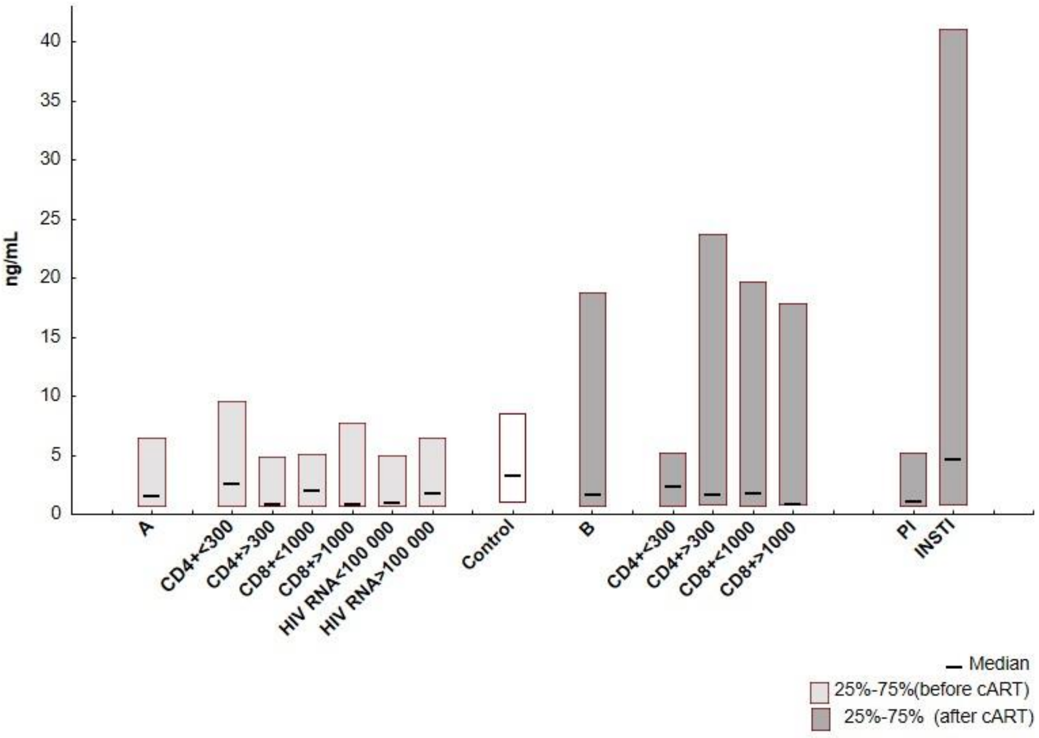
40
35
30
25
20
15
10
5
0
ng/mL
A
CD4+<300
CD4+>300
CD8+<1000
CD8+>1000
HIV RNA<100 000
HIV RNA>100 000
Control
B
CD4+<300
CD4+>300
CD8+<1000
CD8+>1000
PI
INSTI
Median
25%-75%(before cART)
25%-75% (after cART)

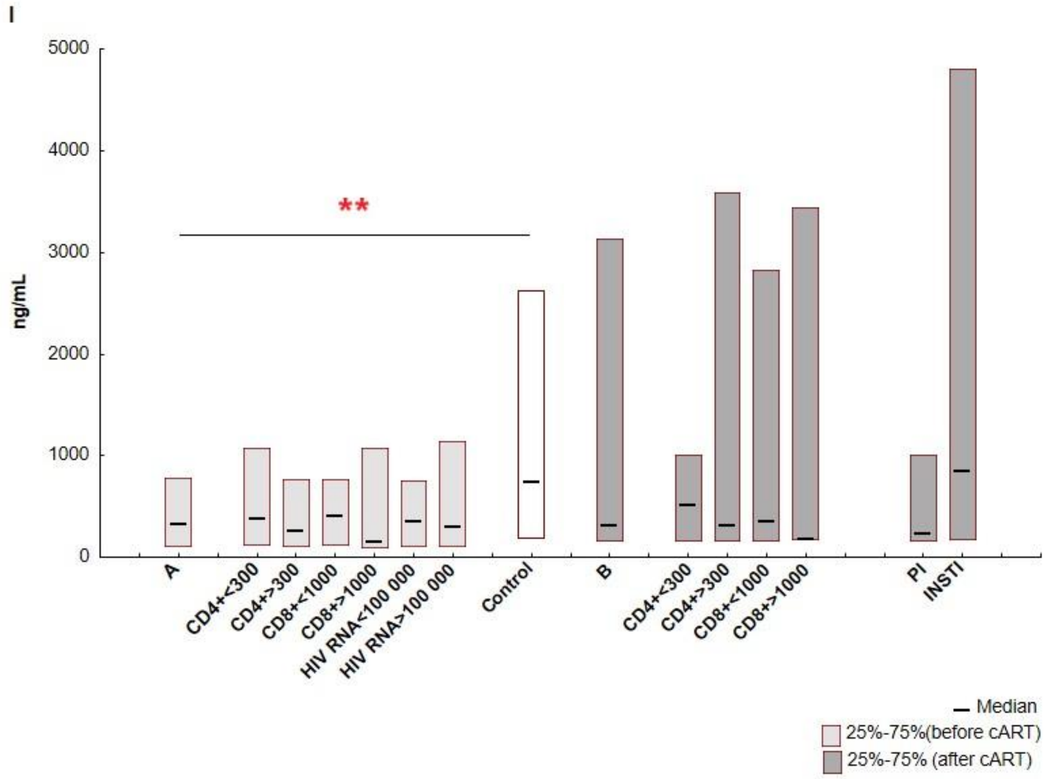

Figure S1. The median and IQR of (**A**) IRS (Irisin), (**B**) MSTN (Myostatin), (**C**) PYY (Peptide YY), (**D**) GLP-1 (Glucagon-like peptide-1), (**E**) DPP-4 (Dipeptidyl peptidase IV), (**F**) FETU-A (Fetuin A), (**G**) PTX3 (Pentraxin 3), (**H**) SDF-1 (Chemokine stromal cell-derived factor 1), (I) RANTES (Regulated on Activation, Normal T Cell Expressed and Secreted) in plasma obtained from HIV-infected men before and after cART depending on the $CD4^+$ and $CD8^+$ T cells count, HIV RNA viral load, the type of therapeutic regimen and control group. A—before cART; B—after cART; PI—protease inhibitors; INSTI—integrase transfer inhibitors; * *p*—statistical significance by Wilcoxon test, $p < 0.05$; ** *p*—statistical significance by Kruskal–Wallis test, $p < 0.05$.
